# A Baby Formula Designed for Chinese Babies: Content Analysis of Milk Formula Advertisements on Chinese Parenting Apps

**DOI:** 10.2196/14219

**Published:** 2019-11-29

**Authors:** Jing Zhao, Mu Li, Becky Freeman

**Affiliations:** 1 School of Public Health Sydney Medical School The University of Sydney Sydney Australia; 2 China Studies Centre The University of Sydney Sydney Australia

**Keywords:** infant formula, food policy, health promotion, marketing, mobile app, parenting, breastfeeding, advertisement

## Abstract

**Background:**

China is the largest market for infant formula. With the increasing use of smartphones, apps have become the latest tool used to promote milk formula. Formula manufacturers and distributors both have seized the popularity of apps as an avenue for marketing.

**Objective:**

This study aimed to identify and analyze milk formula ads featured on Chinese pregnancy and parenting apps, to build the first complete picture of app-based milk formula marketing techniques being used by milk formula brand variants on these apps, and to more fully understand the ad content that potentially undermines public health messaging about infant and young child feeding.

**Methods:**

We searched for free-to-download Chinese parenting apps in the 360 App Store, the biggest Android app store in China. The final sample consisted of 353 unique formula ads from the 79 apps that met the inclusion criteria. We developed a content analysis coding tool for categorizing the marketing techniques used in ads, which included a total of 22 coding options developed across 4 categories: emotional imagery, marketing elements, claims, and advertising disclosure.

**Results:**

The 353 milk formula ads were distributed across 31 companies, 44 brands, and 79 brand variants. Overall, 15 of 31 corporations were international with the remaining 16 being Chinese owned. An image of a natural pasture was the most commonly used emotional image among the brand variants (16/79). All variants included branding elements, and 75 variants linked directly to e-shops. Special price promotions were promoted by nearly half (n=39) of all variants. A total of 5 variants included a celebrity endorsement in their advertising. A total of 25 of the 79 variants made a product quality claim. Only 14 variants made a direct advertisement disclosure.

**Conclusions:**

The purpose of marketing messages is to widen the use of formula and normalize formula as an appropriate food for all infants and young children, rather than as a specialized food for those unable to breastfeed. Policy makers should take steps to establish an appropriate regulatory framework and provide detailed monitoring and enforcement to ensure that milk formula marketing practices do not undermine breastfeeding norms and behaviors.

## Introduction

Despite the strong health message that *breast is best* for growing infants [1-4], and breastfeeding also benefits mothers [5], no country in the world fully meets the recommended breastfeeding guidelines according to a 2017 report by the United Nations International Children’s Emergency Fund and the World Health Organization (WHO) [6]. In the 194 nations evaluated in the report, only 40% of children younger than 6 months are breastfed exclusively, and only 23 countries have exclusive breastfeeding rates above 60% [6]. One of the powerful environmental factors influencing breastfeeding is the ubiquitous presence of breast milk substitutes (BMS) marketing [7-9]. The global milk formula market, which is primarily composed of BMS, reached sales of US $44.8 billion in 2014 and is set to reach US $70.6 billion by 2019 [10]. China’s, including Hong Kong, contribution to the global sales growth was over 50% during 2010 to 2015 [11]. China is the largest market for infant formula, valued at US $17.8 million in 2016 and is projected to more than double in value by 2019 [10].

Omnipresent marketing of BMS negatively affects breastfeeding practices [10]. Examples of this harmful marketing include provision of free products in maternity facilities [12], promotion by health workers [13], and mass media [12] and Web-based advertising [14]. With the increasing use of smartphones, apps have become the latest tool used to promote milk formula. Women utilize pregnancy and parenting apps as primary sources of information and emotional support [15-17]. In China, the number of active users of the most popular parenting app, Babytree, has reached 20 million per month in 2018 [18]. BMS manufacturers and distributors have seized parenting app popularity as an avenue for marketing. In a previous study involving the content analysis of Chinese infant feeding apps, 20 out of 26 apps were found to host infant formula banner ads on their homepages, and 12 apps included e-commerce stores that both sold and advertised infant formula [19]. Although there is emerging research on how milk formulas are being marketed in digital media, little of this research has closely examined apps [9] or focused on China. Given the exponential growth in popularity of pregnancy and parenting apps, there is a need to understand the milk formula marketing techniques on these apps. Equally, although marketing case studies of specific formula companies or specific types of BMS help to highlight the issue in China [20], a more complete picture of milk formula marketing strategies on digital media is needed.

The International Code of Marketing of Breast Milk Substitutes (the Code), published by the WHO in 1981, is an international health policy framework to regulate the marketing of BMS [21]. The Code prohibits all advertising to the public of BMS, including in digital media [21]. The Code recognizes that BMS have a legitimate role to play, when these are necessary. What the Code aims to do is to protect parents from irresponsible and biased marketing of BMS, to make sure that their choices are made based on full, impartial information, rather than misleading, inaccurate, or biased marketing claims, with the goal of protecting breastfeeding and promoting a healthy diet. There should be no advertising or other promotion to the general public of products within the scope of the Code, that is, BMS (any milk formula up to 3 years of age) and pictures or text that idealize the use of BMS should not be used. However, the Code, on its own, is not legally enforceable. The Code states that governments should take action to enforce the principles and aims of the Code, including the implementation of legislation. The Code has been adopted by more than 70% of countries, including China [22]. The Chinese Rules Governing the Administration of Marketing of Breast Milk Substitutes were adopted by 6 government sectors in 1995 [13]. However, driven by commercial interests, China repealed these legal measures in 2016, without any replacement, substantially weakening protection from harmful BMS promotion [21]. The industry uses its immense financial resources to impede country’s efforts to adopt the Code [21] and the money spent on marketing BMS dwarfs that invested by governments promoting breastfeeding [22]. In the United States, for example, BMS companies spend an average of US $30 per infant every year on product promotion, compared with the US $0.21 per baby invested in breastfeeding promotion [23].

Given that this marketing heavily influences infant and young child feeding preferences and choices [10], understanding how all milk formula products are being promoted on pregnancy and parenting apps is essential. The primary aim of this study was to identify and analyze milk formula ads featured on Chinese parenting apps. To begin to build the first complete picture of the milk formula marketing techniques being used by milk formula brands on these apps, in this study, we assessed the range of milk formula brands being advertised on apps, advertising techniques employed by these brands, and the scope of health and nutrition claims embedded in app ads. Our aim was to more fully understand the ad content being deployed by formula brands that potentially undermines public health messaging about infant and young child feeding.

## Methods

### App Selection

Android is the most popular smartphone operating system in China, with an almost two-thirds share of the total market [24]. In February 2018, we accessed the monthly free-download ranking list in the *Pregnancy and Parenting* category of the 360 App Store, the biggest Android app store in China. In that list, apps were ranked by monthly number of times downloaded and all publicly available for free download. We included apps that targeted mothers or mothers-to-be, provided pregnancy and early parenting information, featured tools, like vaccine and pregnancy reminders, and provided support through online social forums. A total of 556 apps were initially included and any apps that had been downloaded below 1000 times per month shown in the ranking list were excluded. We also excluded any apps that were focused on children above 3 years of age; broken or contained a dead link; designed mainly for playing games, reading bedtime stories, singing lullabies, or predicting children’s height; or assisted in choosing a baby name. JZ initially screened each app based on the description page and its associated image in the 360 App store. When JZ was unsure about including a particular app during screening, it was discussed and screened together with ML. The final 79 apps were selected for milk formula ad identification and collection. No ethics approval was required for this study as all data collected were publicly available.

### Advertisement Identification

**Figure 1:**
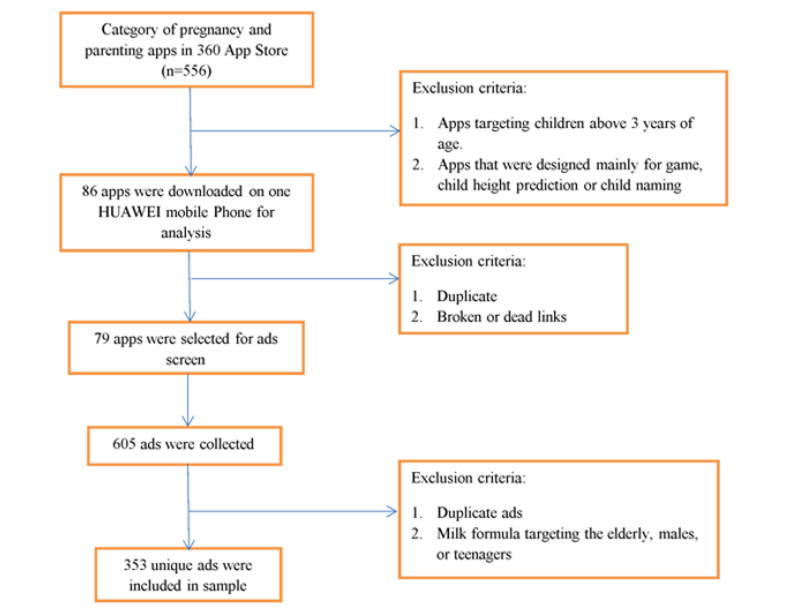
Flow chart for milk formula ads collection.

Ads promoting any type of milk formula were defined as formula ads for this study. Between April and May 2018, we scrolled through the entire app loading page and e-commerce page of each of the included 79 apps to identify and document the formula ads. In addition, as banner ads have been shown to capture audience attention [25], we included any formula ads that appeared on each app opening home page. Initially, all of the formula ads from the selected app pages were captured by screenshot and kept as digital files for further screening analysis. All of the formula ads were then screened, and any duplicate ads and ads not related to milk formula products, such as those for infant food products and milk bottles, were excluded. In addition, any ads for milk formula designed for the elderly, adult men, or teenagers were also excluded. The final sample comprised 353 unique formula ads (Figure 1)**.**

### Advertisement Analysis

Manufacturers frequently offer different variations of the same branded products known as brand variants [26]. In our analysis, we observed that brand variants were very different in terms of name, color, design, options, style, features, and packaging portfolios. Brand variants are used to microsegment consumers with highly differentiated product and also help to ameliorate the effects of marketing restrictions [27]. Therefore, for this analysis, we were most interested in how these brand variants use a range of marketing elements and techniques.

The formula company, brand, and brand variants featured in each ad were identified. Descriptive statistics were used to assess the range of companies, brands, and variants of milk formula, and the frequency of different milk types and target ages. If a single ad promoted more than one type of formula milk or only promoted a company or a brand itself, then that ad was categorized as brand advertising.

On the basis of previous content analysis studies of infant and toddler food ads in magazines [28], food and beverages on Facebook [29], and Web-based advertising of infant formula [30], we developed a content analysis coding tool for categorizing the marketing techniques used in ads in our study. Coding was completed manually by JZ. Initially, this tool was pilot tested on 30 ads randomly chosen from the examined apps. Each of the 30 ads were assessed against the coding tool categories to evaluate appropriateness, completeness, and relevancy to public health communication and also to determine what data would be collected from the ads. After this pilot, we deleted 2 options, *fear emotional appeal* and *vouchers*, *offers, rebates,* and added 2 options, *online shop* and *delivery,* to the coding tool. Finally, a total of 22 coding options were developed across 4 categories, namely, emotional appeal, marketing elements, products claims, and advertisement disclosure (for details, see Table 1).

**Table 1:** Coding categories and options of milk formula ad content.

Coding category and coding options		Definition or examples
**Emotional imagery (based on ad imagery)**		
	Happy child	Image of a smiling baby.
	Parental love	Image of a parent hugging or kissing a baby.
	Satisfied baby drinking from a milk bottle or holding a formula can	Image of a cute baby drinking milk from a bottle or holding a milk formula can.
	Relieved mother, pregnant women	Image of a mother or pregnant woman relieved or at ease.
	Beautiful natural environment, pasture	Image of a mountain pasture, clear sky, and natural landscape.
**Marketing elements**		
	Branding elements	Any logos, colors, and trademarks.
	Special price promotions	Limited-time offers, discount, or other reduced price.
	Sampling	Consumers are given some quantity of a product for no charge (either new or established products).
	Coupons	A document or e-voucher exchanged for financial discount with product purchase.
	Bonus packs	Special packaging that provides consumers with extra quantities of products at no extra charge.
	Competitions, prizes, and giveaways	Any contest involving a participant entry, including content about the product or the brand (such as photos and article) created by users.
	Cartoon characters or spokes characters	Any nonhuman characters that are used to promote a product or a brand—giraffes, sheep, bees, bears, and other cartoon images.
	Sponsorships and partnerships	Any events that the brand supports or other brands with which the brand partners.
	Celebrity endorsement	People with an entertainment or media profile.
	Online shop	The ad is directly linked to the products in e-shop, and app user could buy the product online.
	Delivery	Products are delivered directly to consumer.
**Claims**		
	Food safety claim	Purity, no contamination, imported milk source, good quality of milk source, whole package imported, and a long brand history.
	Product quality claim	Organic, goat milk, premium or gold or super package, scientific based or medical evidence based or medical recommendations based or trusted by parents and health professionals.
	Health claims	Nutritionally balances, and provides good nutrition to, the infant or children, supports healthy growth/better overall health, helps to support digestive system, good for the brain/better brain power, and specialized for allergic baby.
	Nutrition claims	Contains DHA^a^ or ARA^b^, Omega 3, or milk fat globule; contains fiber; contains probiotics; contains protein or amino acids; contains lactose; contains vitamin.
	Claims idealizing the use of milk formula	Texts aim to idealize the use of milk formula, including wording *closest to*, *inspired by*, and *following the example of* human breast milk; *closer to breast milk*.
Advertisement disclosure		All advertising has to be identified with the word *Advertisement*.

^a^DHA: docosahexaenonic acid.

^b^ARA: arachidonic acid.

First, any emotional imagery included in the ads was coded. Emotional images were coded to one of the following 5 options: (1) happy child, (2) parental love, (3) satisfied baby drinking from a milk bottle or holding a formula can, (4) relieved mother, pregnant women, and (5) beautiful natural environment, pasture. Any duplicate images that appeared in multiple ads were not assessed. Next for each ad, the presence of any of the 11 marketing element categories was recorded. Marketing element categories included branding, special price promotions, sampling, coupons, bonus pack, contests, prizes and giveaways, sponsorships, spokes characters and celebrity endorsement, and free delivery. The third coding category, claims, was made up of 5 coding options: food safety claims, product quality claims, health claims, nutrition claims, and claims idealizing the use of formula, with several types of claim under each option. Finally, for the last coding category, disclosure, it was noted whether the ad included text identifying that it was advertising content. According to China’s *Interim Measures for the Administration of Internet Advertising*, which was launched in 2016, all digital advertising must be clearly identified with the word *advertising*. Ads were initially coded using the exact Chinese characters that appeared in ads, and then the coding results are reported here in English.

All content coding was completed by JZ and then tested for accuracy by a research assistant independently coding 40 ads randomly selected from the final sample. Cohen kappa was used to measure intercoder reliability using SPSS version 22.0 statistical software (SPSS Inc, an IBM Company). Kappa value was 0.81. Definitions for infant formula and substitutes is provided in Textbox 1.

**Definitions of infant formula, follow-on formula, and breast milk substitutes.**

- Breast milk substitutes refer to any food for children (up to 3 years of age) being marketed or otherwise presented as a partial or total replacement for breast milk, whether suitable for that purpose or not [21]
- Milk formula refers to the wider range of milk powders for all ages available on the market [21]
- Infant formula refers to milk formula products intended for infants during the recommended exclusive breastfeeding phase (typically 0-6 months of age) [21]
- Follow-on formula refers to milk formula products intended for older infants, as they begin to receive complementary foods, and young children (typically 6-12 months of age) [21]
- Toddler milk: A fortified milk-based product only suitable for children older than 12 months (12-36 months) [21]
- Child milk: A fortified milk-based product only suitable for children older than 36 months [21]

## Results

### General Characteristics

In total, 353 unique ads from the 79 apps were collected for analysis. The 353 milk formula ads were distributed across 31 companies, 44 brands, and 79 brand variants. Just over half of the milk formula companies (15/31) were international corporations with the remaining 16 Chinese-owned. Many of the international companies marketed multiple brands and brand variants with the majority of brands (27/44, 61%) and brand variants (49/79, 62%) being produced by international companies. In all, 70 of the 353 ads were distributed across 10 variants from 4 brands manufactured by the Danone Company. This was followed by Nestle, with 52 ads across 11 variants under 4 brands and then Mead Johnson with 34 ads across 7 brand variants under 6 brands.

More than one-thirds of brand variants (n=28) advertised more than one type of milk formula. Multimedia Appendix 1 shows the full distribution of the types of milk formula across brands and brand variants that appeared in the ads. Infant formula was advertised by 24 brand variants, follow-on formula by 30 brand variants, and toddler milk formula by 40 brand variants. A further 11 brand variants promoted formula for children, and 1 variant included a formula for older children. A total of 10 brand variants were for milk formula that was promoted as being for pregnant and breastfeeding women. Another 3 variants claimed to have formulas that were specifically designed for babies born prematurely. There were 11 brands and 15 variants identified in general brand ads that were not for any specific type of milk formula.

### Emotional Imagery

An image of a natural pasture was the most commonly used emotional image among all 79 brand variants (n=16). Followed by an image of parental love (n=11), happy child (n=7), relieved mother/pregnant women (n=5), and a baby drinking/holding milk bottle (n=4; Table 2).

**Table 2:** Emotional imagery, marketing elements, and advertisement disclosure presented in 79 milk formula brand variant ads.

Coding category and option		Occurrence^a^, n (%)
**Emotional imagery**		
	Beautiful natural environment, pasture	16 (20)
	Parental love	11 (14)
	Happy child	7 (9)
	Relieved mother, pregnant women	5 (6)
	Satisfied baby drinking milk bottle or holding formula can	4 (5)
**Marketing elements**		
	Branding elements	79 (100)
	Online shop	75 (95)
	Special price promotions	39 (49)
	Competitions, prizes, and giveaways	27 (34)
	Delivery	23 (29)
	Coupons	20 (25)
	Bonus packs	17 (22)
	Cartoon characters/spokes characters	16 (20)
	Sampling	14 (18)
	Celebrity endorsement	5 (6)
	Sponsorships and partnerships	4 (5)
Advertisement disclosure		32 (41)

^a^Some brand variants contained more than one emotional imagery or marketing element.

### Marketing Elements

All variants included branding elements, such as logos, trademarks, and brand colors, and linked directly to e-shops. Special price promotions, such as discounts, sales, and limited-time offers, were promoted by nearly half (39/79, 49%) of all brand variants. Competitions, prizes, or giveaways on the condition of supplying contact details were used by 27 variants. A user-generated content (UGC) contest was held by one brand variant (Enfinitas) where consumers were asked to submit a photo of or a story about the milk formula to win a gift. A total of 23 variants offered free delivery service. Coupons, distributed either by the brand or online store, were available in the ads of 20 variants. A total of 17 variants offered a bonus pack and 16 variants included licensed characters to promote their products, such as cartoon giraffes, sheep, bees, and bears. Time-limited product sampling was identified in the ad of 14 variants of both established formulas and new products. A total of 5 variants included a celebrity endorsement in their advertising. These celebrities were all well known in China, included an actress who is a mother and is followed by nearly 30 million fans on the social media site Weibo [31]. In all, 4 brand variants engaged in sponsored activities, such as Wyeth Promama advertised its official mothers’ club, Biostime Supreme sponsored an education activity when traveling with young kids, Nan HA promoted a Web-based parent personality test, and Aptamil German sponsored a TEDx program titled *Trust is visible* in China, where 5 well-known individuals shared their stories about trust and relationships. The brand variant name and logo were all displayed prominently in association with the sponsored activities. Many brand variant ads contained more than one marketing element, and some marketing elements appeared in more than one brand variant advertising, but each brand variant was counted only once for each element (Table 2).

### Claims

Many brand variant ads contained more than one claim across the 5 coding options. Although some type of claims appeared in more than one ad for a single brand variant, each unique type of claim was counted only once (Table 3). A total of 34 brand variants made at least one type of food safety claim. A total of 27 domestic brand variants included at least one food safety claim, with 7 stating to be *100% imported source* and a further 20 highlighting *100% produced and packaged overseas*. Overall, 9 brand variants claimed to use good quality of milk source, and 8 brand variants claimed to have a *long brand history*. At least one type of product quality claim was made by 25 brand variants. The most identified product quality claim among brand variants was *super/gold/premium* (n=20), then *organic* (n=7) and *scientific/evidence based* (n=5), with the claim *goat milk* being used in 4 brand variants. A total of 24 brand variants made at least one health claim, with 8 claiming to improve digestion and absorption, 7 claiming to protect against allergies, 6 claiming to support growth and development/better overall health, and 3 claiming to improve brain development. For the 24 brand variants that made at least one nutrition claim, the phrase “Contains DHA/ARA, Omega 3, or Milk Fat Globule Membrane,” was the most frequently identified claim (n=7), whereas 5 variants highlighted the addition of sphingomyelin or choline; 4 mentioned added protein or amino acids, 4 were said to contain probiotics, 2 claimed to include lactose-containing milk formula, and 1 claim related to each of vitamins and fiber. In the claim category idealizing the use of milk formula, 10 brand variants used words idealizing milk formula and bottle-feeding, including *thousands of mothers’ choice*, *similar to breast milk,* or *love*. Multimedia Appendices 2 and 3 are typical examples of milk formula ads examined in the study.

**Table 3:** Prevalence of 79 milk formula brand variants making claims by type of claim.

Claim type		Brand variant making this claim type^a^, n
**Food safety claims**		34
	Whole package imported	20
	Good quality of milk source	9
	Long brand history	8
	Imported milk source	7
	Purity, no contamination	2
**Product quality claims**		25
	Premium, gold package, super	20
	Organic	7
	Scientific based, medical evidence based/medical recommendations based/trusted by parents and health professionals.	5
	Goat milk	4
**Health claims**		24
	Helps to support digestive and absorption system	8
	Specialized for allergic baby	7
	Nutritionally balances and provides good nutrition to infant or children/supports healthy growth/better overall health	6
	Good for the brain/better brain power	3
**Nutrition claims**		24
	Contains DHA^b^/ARA^c^, Omega 3, or milk fat globule membrane	7
	Contains sphingomyelin or choline	5
	Contains protein or amino acids	4
	Contains probiotics	4
	Contains lactose	2
	Contains fiber	1
	Contains vitamin	1
**Claims idealizing the use of milk formula**		10
	Including *thousands of mothers’ choice*/*similar to breast milk*/*love*	10

^a^Some brand variants made multiple types of claims under each claim option.

^b^DHA: docosahexaenonic acid.

^c^ARA: arachidonic acid.

### Advertisement Disclosure

In total, only 14 variants made a direct advertisement disclosure. In addition, a further 18 variants included the Chinese character for “product/commodity” instead of advertisement. A total of 2 variants (NAN Pro and S-26 Promil Ultima) stated *breast milk is the best* at the bottom of their ads (see Table 3).

## Discussion

### The Influence of Milk Formula Branding on Breastfeeding

This study identified a total of 79 brand variants that included multiple types of milk formula, with 24 of these brand variants advertising infant milk formula, 30 follow-on formula, and 40 toddler formula. This reflects a full-scale breach of the Code, which applies advertising restrictions to any milk specifically marketed for feeding infants and young children up to the age of 3 years. Since 1986, the WHO has maintained the position that follow-on formula is nutritionally unnecessary, but most countries only apply partial advertising restrictions to infant formula [21]. Toddler milk formula is reportedly set to outperform sales of infant and follow-on milk formula [20], which might explain in part our findings that 40 variants advertised toddler milk formula. Manufacturers are continuing to focus on the growth prospects in the toddler milk formula market to widen their consumer base to include older children. Strikingly, we identified 3 brand variants of milk formula marketed specifically for premature babies, which directly contradicts the WHO advice that “preterm birth infants who are able to breastfeed should be put to the breast as soon as possible after birth when they are clinically stable and should be exclusively breastfed until 6 months of age” [32].

In addition, 11 brand variants promoted child (aged >36 months) milk formula, with one brand variant, Enfakid, only promoting child milk formula; the remaining variants advertised more than one type of milk formula. As the Code is only designed to prevent companies from promoting milk formula and food for children up to 3 years of age, these products and promotions skirt advertising regulations. Moreover, China launched new infant milk formula registration measures in 2015 that came into effect on January 1, 2018. This legislation limits the number of brands per manufacturer to 3 for each of the development stages under 3 years of age [33]. This means child milk formula is not explicitly regulated by the new regulation, which may also help explain the 11 brand variants identified in our study that included a child milk formula.

Brand extension and brand-focused advertising help to establish brand awareness, preference, and loyalty to formula from fetus through to early childhood, this includes influencing pregnant women and mothers who are breastfeeding their infants. We found 10 variants of a special type of milk formula marketed as being for breastfeeding/pregnant women. Marketing an increasing range of milk formulas for different age groups weakens the impact of restrictions on infant formula (0-6 months). Sharing a brand that is identified with other life stages of formula is likely to influence infant feeding behavior to the same extent as direct advertising, as consumers are unable to differentiate between the two [34]. Similarly, ads for follow-on formula are perceived by pregnant women and mothers as promoting infant formula [35]. Research carried out in Australia [34], the United Kingdom [36], and Italy [35] showed how difficult it is for pregnant women and mothers to identify the difference between promotion of follow-on or toddler formula and promotion of infant formula. As products in these older age group categories are often branded, packaged, and labeled in ways that resemble infant formula, they can also be erroneously introduced in the first 6 months of life. Consumers then assume that the claims made in these brand variants are also true for the infant formula variants [35].

Manufactures use persuasive marketing techniques to reinforce their brand identity, such as special price promotion, coupons, prizes, and giveaways. This is despite the fact that the Code prohibits any point of sale advertising or any promotional technique to induce sales [21]. Of particular note is the UGC contest, consumers were asked to submit photos and content that expressed their *love* of the promoted milk formula. UGC is a form of peer endorsement that increases brand authenticity and enhances consumer trust. This distinctive social media marketing tactic builds on the effectiveness of competitions by allowing companies and consumers to collaborate and build brands together [35]. The explosion of e-commerce and the ubiquitous promotion and availability of milk formula in the Chinese online environment encourages and enables consumers to directly purchase products within pregnancy and parenting apps. Both domestic and overseas manufacturers have enjoyed strong sales through online shopping [37].

Some brand variants advertised with claims of *love* and *similar to breast milk* in Chinese characters, which could increase exposure to idealized bottle feeding and raise desire to use milk formula. Pregnant women and new mothers exposed to BMS ads containing the words *close* and *similar* believe infant formula is nutritionally equivalent to human milk [38]. This is another clear violation of the Code that states not to contain pictures or text that idealizes the use of BMS. In addition, the most commonly used (by 16 variants) image of *beautiful natural pasture and clear sky* implies the pure source and high quality of milk formula, which plays to Chinese mothers’ food safety concerns around domestic brands [39]. This is due to the 2008 Chinese milk scandal where milk formula was found to contain high concentrations of melamine and was estimated to cause tens of thousands of infants and children to develop severe kidney problems [40].

### The Influence of Marketing Claims on Breastfeeding

The 2008 milk product scandal in China has also led to high demand for imported infant formula. Large international companies exploit these product fears to create a premium image of their brands. Meanwhile, domestic companies are attempting to rehabilitate their brand image while also making similar reassuring claims that they use *100% imported source* or *100% produced and packaged overseas* that are paired with emotional images of nature. For example, the brand variant-NAN pro claimed that its formula is imported from Switzerland and included an image of a snow-capped mountain. Premium brand variants featured heavily in our results. Marketing of so-called premium products with the associated *premium* pricing is leading to confusion among consumers and having a significant financial impact on families, especially in Asian countries [8,11]. A case study from Singapore found that parents perceived that more expensive or premium products are of higher quality, as they lack sufficient understanding of the nutritional content of formula milk [41]. A survey of Chinese bottle-feeding mothers found that 65% of mothers chose organic infant milk because they were willing to pay more for their baby’s food [42].

Heath claims also included the claim of formula with added nutrients and associated benefits. For example, 3 brand variants claimed to contain added ingredients that improve the baby’s intelligence, which may leave some mothers with the impression that their own breast milk is inferior to formula [9]. Again, this is despite the Code clearly stating not to permit the formula to be marketed as being equal to or comparable with breast milk [21]. This violation is not unique to the Chinese market. In the United Kingdom, an analysis of 13 online parental chat rooms found that the single most repeated idea across the sites was that formula was closest to breast milk, a statement that was originated from the Aptamil (Danone) marketing [43]. A qualitative study of 4 focus groups reported that participants found ads confusing in terms of how formula-feeding is superior to, inferior to, or the same as breastfeeding [38]. We identified 7 brand variants that claimed to help preventing infants from developing allergies, and 8 brand variants claimed their products were good for the baby’s digestive health. However, the evidence for these claimed benefits is not established [44]. A US study found that more than half of the 22 infant formula products reviewed were marketed with claims, whereas none of which were backed by publicly available scientific evidence [45].

### Policy Change and Actions Needed

The Chinese national government must take responsibility for ensuring the Code is implemented with adequate enforcement measures. Advertising regulations that restrict a broader range of marketing techniques are urgently needed. Monitoring advertisers’ compliance with such restrictions forms a strong basis for regulation and is particularly important for online and digital media. Moreover, a significant monetary penalty should be applied if milk formula companies or other app platforms are found to be breaking laws. Concurrently, when compared with the innovative strategies used by the milk formula industry, a more active approach is needed to promote breastfeeding by public health authorities, rather than the simple message that breast milk is the best. Scaling up health professional advice to breastfeed alongside engaging media campaigns or social mobilization events such as the national breastfeeding day or world breastfeeding week is crucial. Brazil, for example, is widely recognized for implementing a successful National Breastfeeding Program that has made a substantial improvement in breastfeeding exclusivity and duration [46]. This society-wide program includes regulation of the commercialization of infant formula and foods, a strict enforcement of the Code, training for health workers and the development of mother-to-mother support groups, maternity leave extended to 6 months, introduction of the Baby Friendly Hospitals Initiative, and investment in over 200 human milk banks [46]. Public health professionals should also learn from the milk formula industry in terms of constructing messages and applying proven marketing techniques in promoting breastfeeding. In China in particular, there are few health professional–endorsed parenting apps that advocate for breastfeeding and healthy infant feeding, and the space is primarily occupied by commercial entities [19].

### Strengths and Limitations

A key strength of the study is that we developed and established a comprehensive coding tool to manually analyze milk formula ad content, including text and images, in a non-English language context. This content analysis also has some limitations. First, this study is only a snapshot of a small number of available free-to-download parenting apps. Second, although the ad collection was not confined to one type of milk formula, we may have missed some milk formula brands or variants, as many products are being continually added and removed from the market. Although a second independent coder was used to test the accuracy of the ad coding and high agreement was achieved, it is possible that ad characteristics could be missed or miscoded.

### Conclusions

This is the first study to analyze milk formula ads found on the popular Chinese parenting apps. Products that function as BMS should not be freely advertised [10]. The purpose of these marketing messages is to widen the use of formulas and normalize formula as an appropriate food for all infants and young children rather than as a specialized food for those unable to breastfeed. The present analysis affirms a need for greater efforts in implementation, monitoring, and enforcement of the Code. Implementation of the Code is not possible without adequate funds and allocated budgets from national and local governments. As stated, China adopted the Code in 1995 but repealed these legal measures in 2016. The government should enshrine the Code in law to both ensure effective monitoring and comprehensive enforcement of unethical BMS marketing practices and to create an environment where breastfeeding is normal, accepted, and protected.

## Appendix

Multimedia Appendix 1 Distribution of the types of milk formula across brands and brand variants that appeared in the advertisements.

Multimedia Appendix 2 Example of a2 Platium formula advertisement featuring emotional imagery, marketing elements and claims.

Multimedia Appendix 3 Example of NAN Pro formula advertisement featuring emotional imagery, marketing elements and claims.

## Abbreviations

BMS: breast milk substitutes
Code: The International Code of Marketing of Breast Milk Substitutes
UGC: user-generated content
WHO: World Health Organization

## References

[1] Heinig MJ (2001). Host defense benefits of breastfeeding for the infant. Effect of breastfeeding duration and exclusivity. Pediatr Clin North Am.

[2] Jones G, Steketee RW, Black RE, Bhutta ZA, Morris SS, Bellagio Child Survival Study Group (2003). How many child deaths can we prevent this year?. Lancet.

[3] Newton ER (2004). Breastmilk: the gold standard. Clin Obstet Gynecol.

[4] Quigley MA, Kelly YJ, Sacker A (2007). Breastfeeding and hospitalization for diarrheal and respiratory infection in the United Kingdom Millennium Cohort Study. Pediatrics.

[5] (2000). Effect of breastfeeding on infant and child mortality due to infectious diseases in less developed countries: a pooled analysis. WHO Collaborative Study Team on the Role of Breastfeeding on the Prevention of Infant Mortality. Lancet.

[6] World Health Organization (2017). Babies and mothers worldwide failed by lack of investment in breastfeeding. Saudi Med J.

[7] Mason F, Rawe K, Wright S (2013). Save the Children.

[8] Baker P, Smith J, Salmon L, Friel S, Kent G, Iellamo A, Dadhich JP, Renfrew MJ (2016). Global trends and patterns of commercial milk-based formula sales: is an unprecedented infant and young child feeding transition underway?. Public Health Nutr.

[9] Piwoz EG, Huffman SL (2015). The impact of marketing of breast-milk substitutes on WHO-recommended breastfeeding practices. Food Nutr Bull.

[10] Rollins NC, Bhandari N, Hajeebhoy N, Horton S, Lutter CK, Martines JC, Piwoz EG, Richter LM, Victora CG, Lancet Breastfeeding Series Group (2016). Why invest, and what it will take to improve breastfeeding practices?. Lancet.

[11] (2017). Changing Markets.

[12] Xu F, Liu X, Binns CW, Xiao C, Wu J, Lee AH (2006). A decade of change in breastfeeding in China's far north-west. Int Breastfeed J.

[13] Liu A, Dai Y, Xie X, Chen L (2014). Implementation of international code of marketing breast-milk substitutes in China. Breastfeed Med.

[14] Zhang Y, Carlton E, Fein SB (2013). The association of prenatal media marketing exposure recall with breastfeeding intentions, initiation, and duration. J Hum Lact.

[15] Derbyshire E, Dancey D (2013). Smartphone medical applications for women's health: what is the evidence-base and feedback?. Int J Telemed Appl.

[16] Hearn L, Miller M, Fletcher A (2013). Online healthy lifestyle support in the perinatal period: what do women want and do they use it?. Aust J Prim Health.

[17] Johnson SA (2014). 'Maternal devices', social media and the self-management of pregnancy, mothering and child health. Societies.

[18] (2018). Useit.

[19] Zhao J, Freeman B, Li M (2017). How do infant feeding apps in China measure up? A content quality assessment. JMIR Mhealth Uhealth.

[20] (2016). SlideShare.

[21] World Health Organization, UNICEF, IBFAN (2018). Marketing of Breast-milk Substitutes: National Implementation of the International Code.

[22] Lutter CK (2013). The international code of marketing of breast-milk substitutes: lessons learned and implications for the regulation of marketing of foods and beverages to children. Public Health Nutr.

[23] International Baby Food Action Network.

[24] (2018). Statista.

[25] Sajjacholapunt P, Ball LJ (2014). The influence of banner advertisements on attention and memory: human faces with averted gaze can enhance advertising effectiveness. Front Psychol.

[26] Bergen M, Dutta S, Shugan SM (1996). Branded variants: a retail perspective. J Mark Res.

[27] Greenland SJ (2015). Cigarette brand variant portfolio strategy and the use of colour in a darkening market. Tob Control.

[28] Chen Y, Chang J, Gong Y (2015). A content analysis of infant and toddler food advertisements in Taiwanese popular pregnancy and early parenting magazines. J Hum Lact.

[29] Freeman B, Kelly B, Baur L, Chapman K, Chapman S, Gill T, King L (2014). Digital junk: food and beverage marketing on Facebook. Am J Public Health.

[30] Abrahams SW (2012). Milk and social media: online communities and the International Code of Marketing of Breast-milk Substitutes. J Hum Lact.

[31] Weibo.

[32] Edmond K, Bahl R (2007). World Health Organization.

[33] Chen E (2017). mondaq.

[34] Berry NJ, Jones SC, Iverson D (2012). Toddler milk advertising in Australia: infant formula advertising in disguise?. Australasian Mark J.

[35] Cattaneo A, Pani P, Carletti C, Guidetti M, Mutti V, Guidetti C, Knowles A, Follow-on Formula Research Group (2015). Advertisements of follow-on formula and their perception by pregnant women and mothers in Italy. Arch Dis Child.

[36] Family Health Service, The Department of Health Hong Kong SAR Government (2013). Survey on Mothers' Views of Formula Milk Promotion and Information on Infant and Young Child Feeding.

[37] Mason FG (2018). Save the Children.

[38] Parry K, Taylor E, Hall-Dardess P, Walker M, Labbok M (2013). Understanding women's interpretations of infant formula advertising. Birth.

[39] Ji A, Wong YI, Cai T, Liu J (2014). Infant formula safety concerns and consequences in China. World J Pediatr.

[40] Parry J (2008). China's tainted milk scandal spreads around world. Br Med J.

[41] (2017). The Competition Commission of Singapore.

[42] Cheryl N Mintel.

[43] The Caroline Walker Trust.

[44] Abrams SA (2015). Is it time to put a moratorium on new infant formulas that are not adequately investigated?. J Pediatr.

[45] Belamarich PF, Bochner RE, Racine AD (2016). A critical review of the marketing claims of infant formula products in the United States. Clin Pediatr (Phila).

[46] Pérez-Escamilla R (2017). Breastfeeding in Brazil: major progress, but still a long way to go. J Pediatr (Rio J).

