# Supplementary material for: A Baby Formula Designed for Chinese Babies: Content Analysis of Milk Formula Advertisements on Chinese Parenting Apps
Source: JMIR Mhealth Uhealth. 2019 Nov 29;7(11):e14219. doi: 10.2196/14219 (PMC6911233; doi:10.2196/14219)
Supplement: Multimedia Appendix 1 [file mhealth_v7i11e14219_app1.docx]

| Company Name | Brands | Brand advertising | Brand variants | Types of milk formula advertised | | | | | | | |
| --- | --- | --- | --- | --- | --- | --- | --- | --- | --- | --- | --- |
|  |  |  |  | Brand variant advertising | Infant | Follow-on | Toddler | Child | Older Child | pregnant and/or breastfeeding women | Baby born prematurely |
| **International companies** | | | | | | | | | | | |
| Nestle | Wyeth-S26 |  | Wyeth Promama |  |  |  |  |  |  | X |  |
|  |  |  | Wyeth S-26 Ultima | X |  | X |  |  |  |  |  |
|  |  |  | Wyeth S-26Process |  |  |  | X |  |  |  |  |
|  |  |  | Wyeth S-26 Nutrisure Gold | X |  |  |  |  |  |  |  |
|  | Nan |  | NAN Pro | X |  |  | X |  |  |  |  |
|  |  |  | NAN Gold |  |  |  | X |  |  |  |  |
|  |  |  | NAN HA | X |  | X |  |  |  |  |  |
|  |  |  | NAN Pre |  |  |  |  |  |  |  | X |
|  | Gerber | X | Gerber |  |  |  |  |  |  |  |  |
|  | illuma | X | illuma |  | X | X | X |  |  |  |  |
|  |  |  | Illumcare |  |  |  |  |  |  | X |  |
| Danone | Dumex |  | Dumex |  |  |  | X |  |  |  |  |
|  |  |  | Dumex pregnant |  |  |  |  |  |  | X |  |
|  |  |  | Dumex diamor | X |  | X |  |  |  |  |  |
|  | Aptamil |  | HA Aptamil |  |  |  |  |  |  |  |  |
|  |  |  | Gold Aptamil |  | X |  |  |  |  |  |  |
|  |  |  | Aptamil |  | X |  |  |  |  |  |  |
|  |  |  | Australia Aptamil |  | X |  | X | X |  |  | X |
|  |  |  | German Aptamil | X | X |  | X |  |  |  |  |
|  | Nutrilon |  | Nutrilon |  | X | X | X | X |  |  |  |
|  | Cow&Gate |  | Cow&Gate |  | X | X | X | X |  |  |  |
| MeadJohnson | Enfamil |  | Enfamil |  | X |  |  |  |  |  |  |
|  |  |  | Enfamama |  |  |  |  |  |  | X |  |
|  | Enfagrow |  | Enfagrow | X |  |  | X |  |  |  |  |
|  | Enfinitas |  | Enfinitas | X | X | X | X |  |  |  |  |
|  | Nutramigen |  | Nutramigen |  | X |  |  |  |  |  |  |
|  | Enfakid |  | Enfakid |  |  |  |  | X |  |  |  |
|  | Enfaschool |  | Enfaschool |  |  |  |  |  | X |  |  |
| Abbott | Eleva |  | Eleva |  |  |  | X |  |  |  |  |
|  | Similac |  | similac-NeoSure |  |  |  |  |  |  |  | X |
|  |  |  | similac |  |  |  | X | X |  |  |  |
|  |  |  | Similac pregnant |  |  |  |  |  |  | X |  |
| Friesland Campina | Frisola |  | Friso |  |  |  | X | X |  |  |  |
|  |  |  | Gold Friso |  |  |  | X |  |  |  |  |
|  |  |  | Frisolac |  | X |  |  |  |  |  |  |
| Hipp | Hipp |  | hipp |  | X | X |  |  |  |  |  |
|  |  |  | Hipp Combiotic |  | X | X |  |  |  |  |  |
| Ausnutria Nutrition | Kabrita |  | Premium Kabrita |  |  |  | X |  |  |  |  |
|  |  |  | Gold Kabrita | X | X | X | X |  |  |  |  |
|  |  |  | Kabrita pregnant |  |  |  |  |  |  | X |  |
| Fonterra | Anmum |  | Anmum |  |  | X | X |  |  |  |  |
|  |  |  | Anmum Pregnanpro |  |  |  |  |  |  | X |  |
| Melin | Mellin |  | Mellin |  |  | X |  |  |  |  |  |
| a2 Milk company | a2 |  | a2 Platinum | X | X | X | X | X |  |  |  |
| Aspen | Alula | X | Alula |  |  |  | X |  |  |  |  |
| Hanmi | Ofmom | X | Ofmom |  |  |  | X |  |  |  |  |
| Bellamys organic | Bellamy |  | Bellamy |  |  | X |  |  |  |  |  |
| Topfer | Topfer |  | Topfer |  |  |  | X |  |  |  |  |
| Nature's One | Baby's only |  | Baby's only |  |  |  | X |  |  |  |  |
| **Chinese companies** | | | | | | | | | | | |
| Sanyuan | ilactou | X | Ilactou |  |  | X | X |  |  |  |  |
|  |  |  | ilactou pregrant |  |  |  |  |  |  | X |  |
|  | Enberre |  | Enberre |  | X | X |  |  |  |  |  |
| Hunan Yahua Dairy | Avadairy | X | Gold Avadariy |  |  | X | X |  |  |  |  |
|  |  |  | By wise Avdariy |  |  | X | X |  |  |  |  |
| Health and Happiness | Biostime | X | π-star |  | X | X |  |  |  |  |  |
|  |  |  | β-star |  |  | X | X |  |  |  |  |
|  |  |  | Supreme |  |  |  | X |  |  |  |  |
| Beingmante Baby & Child Food Co | Beingmate |  | Aijia |  | X |  | X |  |  |  |  |
|  |  |  | Tongxiang |  | X | X |  |  |  |  |  |
|  |  |  | Pregnant |  |  |  |  |  |  | X |  |
| Feihe | Firmus | X | Xingjieyouhu |  |  |  |  |  |  |  |  |
|  |  |  | Feifan |  |  | X |  |  |  |  |  |
|  |  |  | Astrobaby | X |  |  | X |  |  |  |  |
|  |  |  | Organic Zhichun |  |  | X | X |  |  |  |  |
| Yili | YI LI PRO-KIDO |  | PRO-KIDO | X |  |  | X |  |  |  |  |
|  |  |  | PRO-KIDO pregrant |  |  |  |  |  |  | X |  |
| Wandersun | Wandersun | X | ANLICONG |  | X | X | X |  |  |  |  |
|  |  |  | Goldyuanru |  | X | X | X | X |  |  |  |
| Junlebao Dairy Co | Junlebao |  | Super-gold Junlebao | X | X | X | X | X |  |  |  |
| Synutra | Super |  | Super Briffney | X |  |  |  | X |  |  |  |
|  |  |  | Super |  |  | X | X |  |  |  |  |
|  |  |  | SUPER (French version) |  | X |  |  |  |  |  |  |
| Mengniu | Mengniu |  | Mengniu Baijinjiazhi |  |  |  | X |  |  |  |  |
| Baiyue Dairy Group | Yubao | X | Yubao Zhen |  | X | X |  |  |  |  |  |
| Wissun | Wissun |  | Wissun |  |  |  | X |  |  |  |  |
| Engnice | Engnice |  | Engnice |  |  |  | X |  |  |  |  |
| Happy Prince | Happy Prince |  | Happy Prince Zhiai | X |  | X |  |  |  |  |  |
| Fineboon | Fineboon |  | Gold Fineboon |  |  | X |  | X |  |  |  |
| Happy goat | Happy goat | X | Happy goat |  |  |  | X |  |  |  |  |
| **Total** | | | | | | | | | | | |
| **31** | **44** | **11** | **79** | **15** | **24** | **30** | **40** | **11** | **1** | **10** | **3** |
