# Supplementary figures and images for: A Baby Formula Designed for Chinese Babies: Content Analysis of Milk Formula Advertisements on Chinese Parenting Apps
Source: JMIR Mhealth Uhealth. 2019 Nov 29;7(11):e14219. doi: 10.2196/14219 (PMC6911233; doi:10.2196/14219)

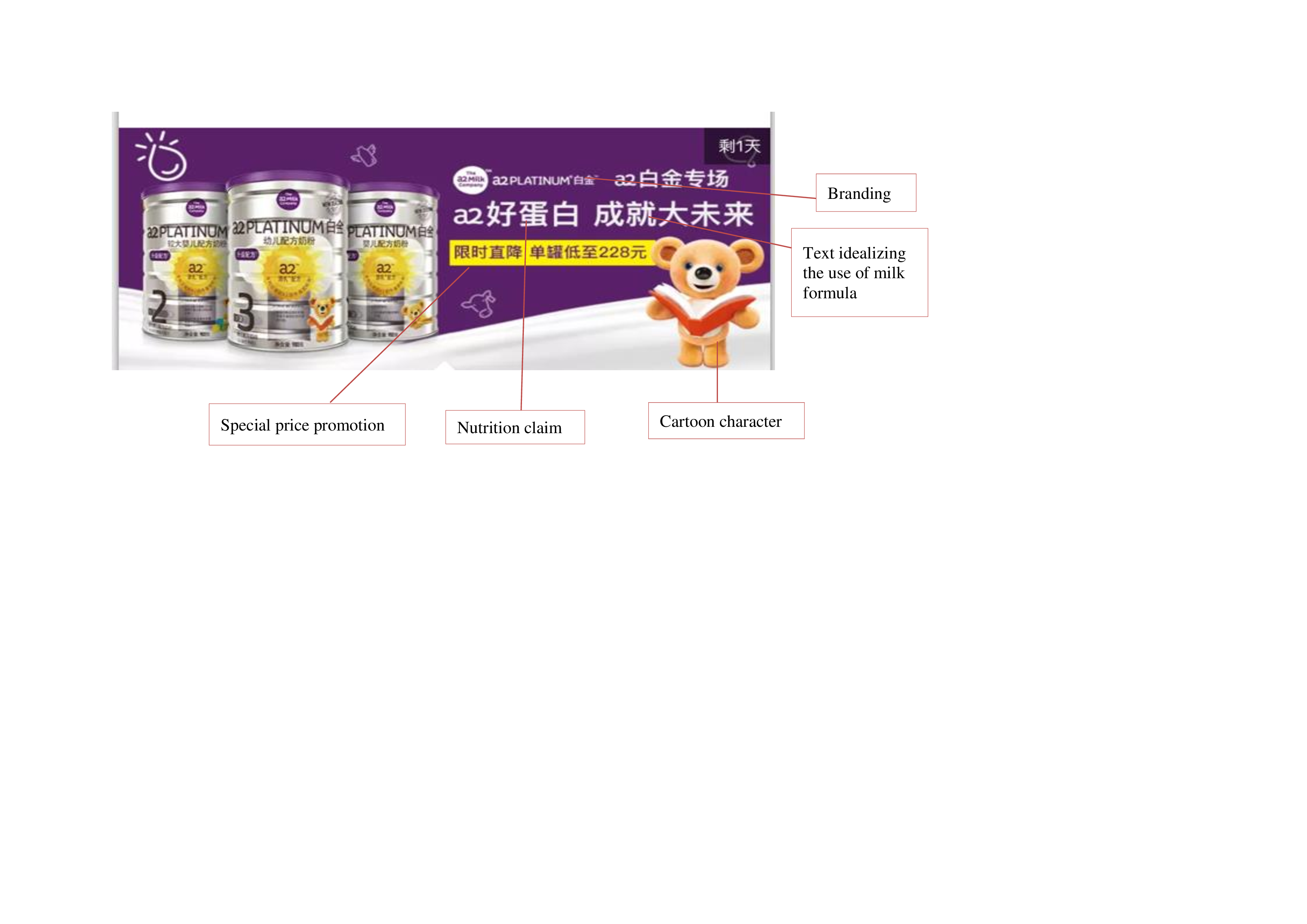

Supplement: Multimedia Appendix 2 [file mhealth_v7i11e14219_app2.png]

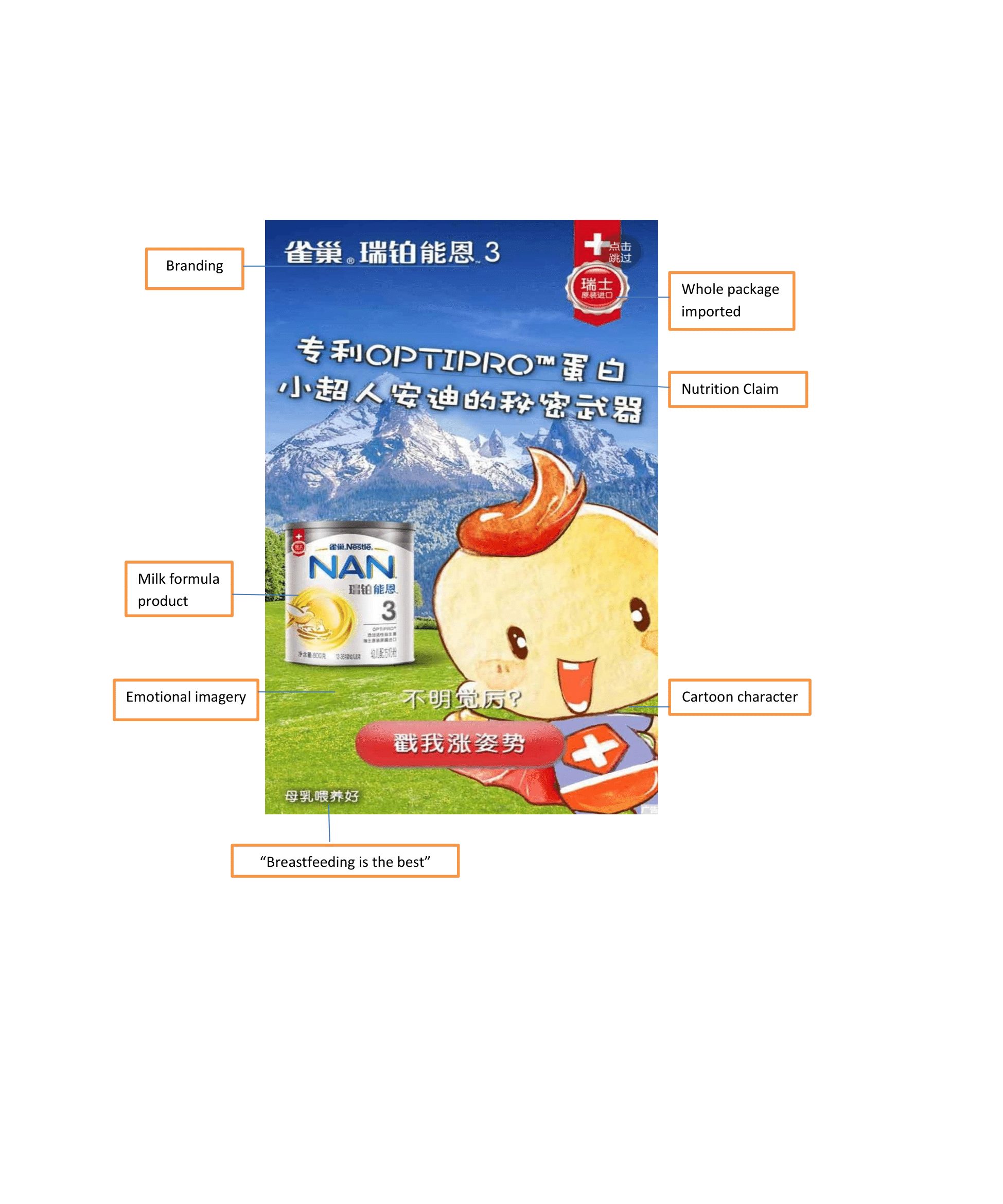

Supplement: Multimedia Appendix 3 [file mhealth_v7i11e14219_app3.png]
